# GERD in elderly patients: surgical treatment with Nissen-Rossetti laparoscopic technique, outcome

**DOI:** 10.1186/1471-2482-12-S1-S4

**Published:** 2012-11-15

**Authors:** Giovanni Aprea, Antonio Ferronetti, Alfonso Canfora, Fabrizio Cardin, Antonio Giugliano, Francesco Guida, Antonio Braun, Melania Battaglini Ciciriello, Federica Tovecci, Giovanni Mastrobuoni, Bruno Amato

**Affiliations:** 1 Department of General, Geriatric, Oncologic Surgery and Advanced Technologies, University “Federico II” of Naples, Via Pansini, 5 - 80131 – Naples, Italy; 2 Department of Surgical and Gastroenterological Sciences, Padova University Hospital, Italy, Via Giustiniani n.2, 35126 Padova, Italy

## Abstract

**Background:**

The gastro-esophageal reflux disease (GERD) is one of the most frequent disease of the upper gastro-entheric tract. Surgical treatment is reserved to selected patients, affected by severe forms of disease and/or without compliance to medical therapy.

In 95%-60% of the patients submitted to surgical antireflux intervention, a notable improvement of the quality of life is observed.

Functional evaluations performed on pre and post – surgical pHmetric and manometric examination have provided new acquisitions about improvements in the restoration of anatomical and functional integrity of the esophagus-gastric antireflux barrier.

**Methods:**

45 elderly patients with GERD were recruited in a 27 months period. All patients were subjected to laparoscopic Nissen-Rossetti 360° fundoplication. The subjects had a pre-surgical evaluation with:

• 24 hours pHmetry,

• esophageal manometry,

The same evaluation was repeated 1 month and 6 months after surgical intervention.

**Results:**

In our series all patients get benefit from surgical treatment, with an improvement of pHmetric and manometric parameters and a regression of complications of GERD such as Barrett's metaplasia. In 8.33% of patients a PPI therapy was necessary, after the surgical intervention, to control symptoms.

**Conclusions:**

The role of surgery in GERD concerns selected patients. Nissen-Rossetti mini-invasive approach is performed with an acceptable percentage of complications (3%-10%). This technique is associated with a good control of GERD symptoms in a short and middle term and with an improvement of functional parameters, such as pHmetric and manometric.

## Background

GERD represents the most frequent disease of the upper gastro-enteric tract: the prevalence in elderly patients is 10%-20% [1,2]. The incidence is around 5 new cases for 1000 people every year, without differences for sex; besides, the disease more frequently appears in elderly patients [3] and in association with obesity and smoke [4-7].

Patient with evidence of severe esophageal lesions (ulcerations, stenosis or Barrett metaplasia) and incomplete symptomatological resolution or recurrence under medical therapy, patient with long duration symptoms or those of young age in which the symptoms persists, should be considered for surgical intervention [8-12].

In the 90%-95% of the patients submitted to surgical antireflux intervention , the resolution of heartburn and regurgitation with notable improvement of quality of the life is observed [11,12]. The answer of the extra-esophageal symptoms to the surgery varies from 60% to 80%. In general, 3-10% of patients shows complications; a lot of complications are minimal and related to surgical interventions, others are more specifically reported to the techniques or to the approach, such as post-operative disphagy (reported in 20% of the patients) [12].

## Methods

The objective of this longitudinal study is to evaluate the effectiveness of videolaparoscopic 360° Nissen-Rossetti fundoplication, in the resolution of GERD related symptoms and in the restoration of anatomical and functional integrity of esophagus-gastric antireflux barrier, in elderly patients, who are elegible of mininvasive surgery, due to their comorbidity.

We conducted a prospective study in a period from September 2009 to January 2012, on 45 elderly patients, treated with VLP Nissen-Rossetti fundoplication. Of these, 36 (80%) completed the follow up to six months, while 9 (20%) have not respected the post-operative protocol, underlining a scarce compliance to undergo invasive examinations. The people that have not completed the first and/or the second post-surgical control have been excluded from the study.

The inclusion criteria for the study were:

1. Patient age > 65

2. Long-term diagnosis of GERD

3. Refractory or intolerated symptoms with conservative treatment, presence of GERD’s atypical symptoms or complications, non-compliance to a long term conservative therapy

4. Absence of other functional esophageal disorders and normal esophageal motility

5. 24 hours pH-metry and/or esophageal manometry suggestive of GERD

6. Comorbidities as cardiovascular disease, diabetes, respiratory disease, etc.

The patients have been preliminarily submitted to a 24 hours pH-metry and to an esophageal manometry; before pHmetric and manometric examinations, patients had to suspend at least for a week the assumption of antisecretive and antiacid drugs, and stop the assumption of prokinetic drugs and medicines that could interfere with the basal tone of the LES (nitrates, calcium-antagonists, aminophylline, beta2-agonists, etc) at least 24 hours before the investigations [9,13,14]. The study foresaw a follow up of six months, that consisted in the repetition of 24 hours pH-metry and manometry one month and six months after the surgical intervention.

Among the different parameters evaluated as functional examinations employed in the study, the ones selected to evaluate the effectiveness of antireflux intervention were:

• for the esophageal manometry: relatively to lower esophageal sphincter (LES), the basal LES tone, whose normal value is > 10 mmHgs and the percentage of relaxation, whose normal value is > 85%; relatively to peristalsis, the ampleness of the peristaltic wave, whose normal interval is between 30 and 180 mmHgs;

• for the 24 hours pH-metry: the acid refluxes number, the esophageal acid exposure time fraction (pH < 4), whose normal value is inferior to 4%, and finally the correlation between episodes of reflux and symptoms, valued with SAP (Symptom Association Probability), that assumes a meaningful value when it is superior to 95%.

On pre-operative and post-operative pHmetric and manometric parameters we applied t-Student test for coupled data, with n-1 degrees of freedom (DoF 12-1 =11) and a P value < 0.05

## Results and discussion

Among the 36 patients evaluated, 21 of them were male (58.3%), while 15 female (41.7%), reflecting a substantial homogeneity in disease distribution in the two sexes (Table 1).

**Table 1:** Sex and middle age of sample

Sex	N° of patients	Middle age
M	21	71.57

F	15	69.79

Total	36	70.83

The age range varied between 65 and 76 years, with a 70.83 years middle age. The middle age for men was of 71.57 years; contrarily the average age for women was of 69.79 years (Table 1). Among the 36 patients, 3 of them (8.33%) were affected by Barrett’s metaplasia.

As the results shown in tables 2 and 3, the LES tone middle value at pre-operative manometry was 15.46 mmHgs, with a standard deviation of 13.49; at the first post-surgical control such parameter had a middle value of 25.33 mmHgs (statistically meaningful increase of 63.84%, in comparison to the initial middle value, P = 0.042), with a standard deviation of 1.86; after 6 months from intervention the LES tone middle value was 25.59 mmHgs (statistically meaningful increase of 65.52% in comparison to the initial value, P = 0,038; not statistically meaningful increase of 1.03% in comparison to middle value after one month, P = 0.20), with a standard deviation of 1.87.

**Tab.2:** Middle values, standard deviations, percentage of variations concerning the parameters evaluated in the study

Parameter	Pre-surgical middle value	Pre-surgical Standard deviation	One month-post-surgical middle value	One month-post-surgical standard deviation	Variation (%)	Six months-post-surgical middle value	Six months-post-surgical standard deviation	Variation (%)
**LES tone (mmHgs)**	15.46	13.49	25.33	1.86	↑ 63.84%	25.59	1.87	↑ 65.52% and ↑ 1.03% (than past control)

**LES relaxation (%)**	107.6	38.74	85.73	24.5	↓ 20.33%	82.95	22.35	↓ 22.91% and ↓ 3.24% (than past control)

**Peristalsis Amplitude (mmHgs)**	91.25	33.60	73.65	17.28	↓ 19.29%	72.61	17.51	20.43% and ↓ 1.41% (than past control)

**N° of acid refluxes**	149.54	65.54	88.27	33.26	40.97%	85.27	30.96	↓ 42.97% and ↓ 3.4% (than past control)

**Esophageal acid exposure fraction of time pH<4 (%)**	10.25	5.68	3.81	2.63	↓ 62.83%	3.34	1.18	67.41% and ↓ and 12.34% (than past control)

**SAP (%)**	96.35	25.52	54.28	28.12	↓ 43.66%	60.44	24.45	37.27% and ↑ 11.35% (than past control)

**Tab.3:** t-Student test for coupled data with n-1 degrees of freedom (DoF 12-1 =11) and P value < 0,05

***t-Student***	Pre-surgical value vs one month-post-surgical values	Pre-surgical value vs six months-post-surgical values	One month-post-surgical values vs six months-post-surgical values
**LES tone**	*P*=0.042	*P*=0.038	*P*=0.20

**LES relaxation**	*P*=0.032	*P*=0.029	*P*=0.28

**Peristalsis amplitude**	*P*=0.041	*P*=0.038	*P*=0.37

**N° of acid refluxes**	*P*=0.026	*P*=0.015	*P*=0.52

**Esophageal acid exposure fraction of time**	*P*=0.019	*P*=0.004	*P*=0.46

**SAP**	*P*=0.011	*P*=0.01	*P*=0.13

About LES relaxation, pre-operative middle value was 107.6% with a standard deviation of 38.74; at the first control the middle value was 85.73% (statistically meaningful decrease of 20.33%, P = 0.032), with a standard deviation of 24.5; at the second control the middle value was 82.95% (statistically meaningful decrease of 22.91% in comparison to the pre-operative value, P = 0.029; not statistically meaningful decrease of 3.24% in comparison to the middle value of first control, P = 0.28), while the standard deviation was 22.35.

Considering peristalsis amplitude, pre-surgical middle value was 91.25 mmHgs with a standard deviation of 33,60; at the first post-operative control such middle value resulted 73.65 mmHgs (statistically meaningful decrease of 19.29%, P = 0.041), with a standard deviation of 17.28; at the second control the middle value for such parameter was 72.61 mmHgs (statistically meaningful decrease of 20.43% in comparison to the initial value, P = 0.038; not statistically meaningful decrease of 1.41% in comparison to the preceding examination, P = 0.37), with a standard deviation of 17.51.

Concerning pHmetric parameters, the pre-operative middle number of acid refluxes in the sample was 149.54, with a standard deviation of 65.54; one month after surgical intervention the middle number of acid refluxes was 88.27 (statistically meaningful decrease of 40.97%, P = 0.026), with a standard deviation of 33.26; after six months the middle number of acid refluxes was 85.27 (statistically meaningful decrease of 42.97% in comparison to the pre-surgical values, P = 0.015; not statistically meaningful decrease of 3,4% in comparison to the middle value after one month from the intervention, P = 0.52) with a standard deviation of 30.96.

The pre-operative middle value of esophageal acid exposure fraction of time was 10.25%, with a standard deviation of 5.68; one month after surgical intervention the middle value was 3.81% (statistically meaningful decrease of 62.83%, P = 0.019), with a standard deviation of 2.63; after six months, instead, the middle value resulted 3.34% (statistically meaningful decrease of 67.41% in comparison to initial middle value, P = 0,004; not statistically meaningful decrease of 12.34% in comparison to the middle value after one month from the intervention, P = 0.46) with a standard deviation of 1.18.

Relatively to pre-operative middle value of SAP, it resulted 96.35%, with a standard deviation of 25.52; at the first control the middle value of SAP was 54.28% (statistically meaningful decrease of 43.66%, P = 0.011), with a standard deviation of 28.12, at the second control the middle value was 60.44% (statistically meaningful decrease of 37.27% in comparison to the initial middle value, P = 0.01; not statistically meaningful increase of 11.35% in comparison to the measured value after one month from intervention, P = 0.13) with a standard deviation of 24.45.

## Conclusions

As already affirmed in other studies, Nissen-Rossetti fundoplication seems to offer indeed a good control of GERD in a short and middle term evaluation, better than other antireflux surgical techniques.

This prospective study has been conducted with the purpose to appraise the real effectiveness of surgical procedure in functional integrity restoration of esophagus-gastric junction, in elderly patients with refractive symptoms to medical therapy. The results of this study show that:

1. In 92,67% of patients submitted to Nissen-Rossetti fundoplication, pH-metric parameters vary with a return within physiological range (reduction of: acid refluxes number, fraction of time of acid esophageal exposure and SAP);

2. The LES basal tone is improved in comparison to the pre-operative period, in all patients.

3. The LES relaxation is decreased, however in the normal range, in comparison to the pre-surgical values, in all patients.

4. In 83.34% of patients, peristalsis is reduced in the post-surgical period, preserving values in normal range.

5. In 100% of patients affected by Barrett’s metaplasia (3 patients), the surgical intervention caused the regression of metaplasia.

6. In 8.33% of patients a PPI therapy was necessary, after the surgical intervention, to control gastric acid secretion and reflux episodes.

From our study, laparoscopic surgical intervention, performed with Nissen-Rossetti technique, gives a statistically meaningful improvement, considering functional parameters, such as pHmetric and manometric. There are not appreciable differences, instead, between parameters evaluated between, one month and six months after intervention, probably because anatomo-functional improvements of the gastro-esophageal tract are appreciable since the immediate post-operative period. Staying our evidences to confirm, we have programmed the prosecution of our experimental study for the next two years, with the intent to reach a sample of subjects such to be able to get more meaningful conclusions concerning the functional results of surgical intervention.

## List of abbreviations

GERD: Gastro-esophageal reflux disease; LES: lower esophageal sphincter; VLP: videolaparoscopy.

## Competing interests

The authors declare that they have no competing interests.

## Authors' contributions

GA, BA: conception and design, interpretation of data, given final approval of the version to be published, AG, AC, AF, AB, MBC, FT, GM,FG: acquisition of data, drafting the manuscript, given final approval of the version to be published, FC: acquisition of data, drafting the manuscript, given final approval of the version to be published.
